# Genome-Wide Analysis of the Cyclin Gene Family in Tomato

**DOI:** 10.3390/ijms15010120

**Published:** 2013-12-23

**Authors:** Tingyan Zhang, Xin Wang, Yongen Lu, Xiaofeng Cai, Zhibiao Ye, Junhong Zhang

**Affiliations:** Key Laboratory of Horticultural Plant Biology, Ministry of Education, Huazhong Agricultural University, Wuhan 430070, China; E-Mails: zhangtingyan86@hotmail.com (T.Z.); wangxinbio@gmail.com (X.W.); luyongen@mail.hzau.edu.cn (Y.L.); cxf0012@163.com (X.C.); zbye@mail.hzau.edu.cn (Z.Y.)

**Keywords:** cyclin, tomato, phylogenetic analysis, gene duplication, gene expression, gibberellin

## Abstract

Cyclins play important roles in cell division and cell expansion. They also interact with cyclin-dependent kinases to control cell cycle progression in plants. Our genome-wide analysis identified 52 expressed cyclin genes in tomato. Phylogenetic analysis of the deduced amino sequences of tomato and *Arabidopsis* cyclin genes divided them into 10 types, A-, B-, C-, D-, H-, L-, T-, U-, SDS- and J18. Pfam analysis indicated that most tomato cyclins contain a cyclin-N domain. C-, H- and J18 types only contain a cyclin-C domain, and U-type cyclins contain another potential cyclin domain. All of the cyclin genes are distributed throughout the tomato genome except for chromosome 8, and 30 of them were found to be segmentally duplicated; they are found on the duplicate segments of chromosome 1, 2, 3, 4, 5, 6, 10, 11 and 12, suggesting that tomato cyclin genes experienced a mass of segmental duplication. Quantitative real-time polymerase chain reaction analysis indicates that the expression patterns of tomato cyclin genes were significantly different in vegetative and reproductive stages. Transcription of most cyclin genes can be enhanced or repressed by exogenous application of gibberellin, which implies that gibberellin maybe a direct regulator of cyclin genes. The study presented here may be useful as a guide for further functional research on tomato cyclins.

## 1. Introduction

Cell cycle regulation is of pivotal importance for plant growth and development. Cell cycle progression is primarily driven by a family of cyclin-dependent kinases (CDKs) in plants. Catalytic activities of CDKs are directly regulated by binding and activation of cyclins, and can be further controlled by several additional mechanisms, including protein phosphorylation/dephosphorylation, proteolysis, and CDK inhibitor protein binding [1–4]. Some studies have also indicated that cyclins contribute to the level of subcellular localization [5].

The first plant cyclin was discovered in soybean in 1991 [6]. Since then, many plant cyclins have been isolated from various plant species. There are 49 cyclins in *Arabidopsis*, which can be divided into 10 types on the basis of function and sequence analysis, including A- to D-type, H-, L-, T-, U-, SDS-, and J18-type [3]. F-type cyclins were reported in rice and suggested to be monocotyledon plant specific [4]. In addition, Q- and Z-types were reported in poplar and defined as new putative cyclin types [7]. According to their expression phase in the cell cycle, cyclins can be divided into two types, M- and G1-cyclins. M-cyclins, including the A- and B-cyclins, help to drive cells into M-phase. G1-cyclins, such as cyclins C, D and E, among others, become active towards the end of the G1-phase and are responsible for ushering the cell into S-phase [8].

It has been shown that a typical cyclin contains a conserved region called the cyclin core, which consists of two domains: cyclin N and cyclin C [9]. The cyclin N domain is also known as the cyclin box and is highly conserved, whereas, the cyclin C domain is less conserved. Some cyclins only contain a cyclin N domain but no cyclin C domain [10], which indicates that the cyclin C domain may not be critical for its function.

Cyclin genes showed different expression patterns during the cell cycle, reflecting their various putative functions. Plant A- and B-type cyclins, known as mitotic cyclins, show a preferential expression pattern during mitosis; A-type cyclins are mainly expressed at the G1/S boundary stage, and combined with Cdk2, they can form a cyclin A/Cdk2 complex, which is required for S-phase transition and DNA replication control.

Three A-type cyclins from tomato were characterized through auxin treatment experiments and provide new insights into cell cycle progression. B-type cyclin genes are expressed only within a narrow time window, from late G2 to mid M phase, and they contribute greatly to the G2/M transition [11–13]. In tomato, suppressed expression of *SlCycB2* through RNAi significantly decreased the number of type I trichomes, indicating its involvement in the regulation of the trichome types [14]. Plants possess a higher complexity of A- and B-type cyclins, and much research has shown that they have extensive and complex functions. D-type cyclins were proposed to be environmental sensors and can trigger the G1/S transition through activation of the RBR/E2F-DP pathway [15–17]. Three D cyclin genes were isolated from young tomato fruit, the D3 cyclin is probably involved in transducing signals leading to fruit growth by cell divisions [18]. Several studies have verified that A-, B-, and D-type cyclins are critical for the mitotic cell cycle and mitotic growth. Few studies have focused on the functions of C-, H-, L-, T-, U-, and SDS-type cyclins; one report on *dsCYC2*, encoding a C-type cyclin in *Phaeodactylum tricornutum* [19], which displayed a light-dependent transcriptional pattern at the G1 checkpoint was found.

Phytohormones are key regulators in plant growth and development. Gibberellins (GAs) are one kind of phytohormones that play a central role in the regulation of growth and development with respect to environmental variability. The roles of GAs in controlling cell division and cell proliferation have been previously extensively documented [20–22].

Tomato is an important fruit plant that serves as a model system for carrying out functional genomics and investigating epigenetic regulation. However, although some tomato cyclins have been reported, genome-wide identification and phylogenetic analysis of the tomato cyclin family have not been reported. Recently, the whole genome sequence of tomato has been published, which provided an excellent opportunity for extensive study of tomato cyclins [23].

Here, we identify 52 cyclin genes in the tomato genome and report on a comprehensive protein sequence analysis, phylogenetic construction, chromosome distribution, and gene structure and duplication analysis. Tissue-specific and GA responsive expression patterns were also examined through quantitative real-time polymerase chain reaction analysis method. These results present a solid foundation for future cloning and functional analysis of tomato cyclin genes.

## 2. Results

### 2.1. Identification of Tomato Cyclin Gene Family

To identify cyclin genes in the tomato genome, keyword searches and sequence alignment were performed against SGN, NCBI, DFCI and other public databases. After removing the redundant sequences, a total of 52 predicted tomato cyclins and/or homologues were identified in Table S1. Some A-, B- and D-type cyclins have been published previously (Table S2), unnamed tomato cyclin genes were named here according to their similarities with *Arabidopsis* cyclins (Table S3). The length of tomato cyclin proteins identified in this study ranges from 142 to 739 amino acids (aa) with an average of 343 aa. The SlCycB2;3 (142 aa) is the smallest tomato cyclin protein, wherein the cyclin domain appears to be truncated at the *C*-terminal end. SlCycB3;1 is the largest tomato cyclin protein (739 aa) and contains two cyclin domains. These genes were distributed on 11 tomato chromosomes, but mainly concentrated on chromosome 4 (9 members), rarely on chromosomes 5 and 9 (2 members) and absent from chromosome 8. All predicted genes and related information are listed in Table 1 including gene names, sequenced IDs, chromosome locations and protein length.

### 2.2. Phylogenetic Analysis of Cyclin Family

**Figure 1.**
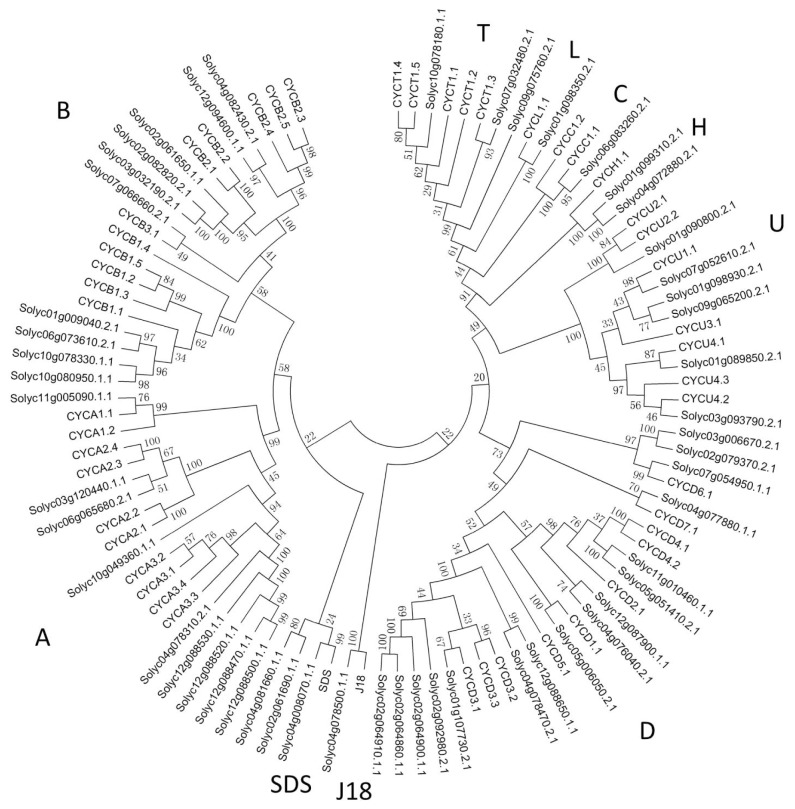
NJ tree of Tomato and *Arabidopsis* cyclins. The cyclins with “Soly” designations are from tomato. The remaining cyclins are from *Arabidopsis*.

To gain an understanding of the relationship between tomato and *Arabidopsis* cyclins, phylogenetic analysis was performed and an N-J phylogenetic tree including 52 tomato and 49 *Arabidopsis* cyclins was constructed (Figure 1). Consistent with the result of *Arabidopsis*, tomato cyclins can be grouped into 10 types, including A-, B-, C-, D-, H-, L-, T-, U-, J18- and SDS- (Solo Dancers) type, and the number of each type was 9, 12, 1, 16, 2, 1, 3, 6, 1 and 1, respectively. For A-, C-, U- and T-type, the numbers of tomato cyclins was 1, 1, 1 and 2, respectively, less than that of Arabidopsis; whereas the numbers of B-, H- and D-type was 1, 1, and 6 more, respectively, when compared with *Arabidopsis*. In addition, both tomato and *Arabidopsis* possess only one member of L-, SDS- and J18-type cyclins. A- and B-type tomato cyclins were more closely related to each other than to other types. T-, L- and H-type formed an independent clade. There were 16 members in D-type cyclins, which formed the largest cluster in tomato cyclin family. U-type cyclins formed a separate clade and all of them just contain one cyclin domain which was predicted to play a role in phosphate signaling.

### 2.3. Structure and Protein Sequence Analysis of Tomato Cyclins

**Figure 2.**
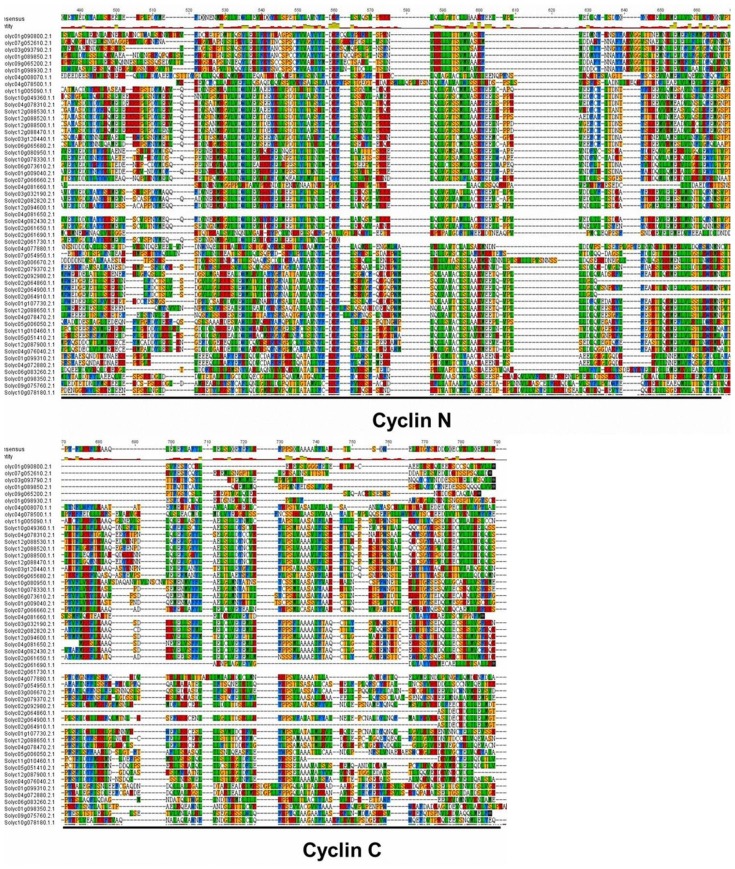
Alignment of cyclin domains. Gene identity numbers are provided on the left. Color shading indicates types of amino acid residues conserved. The defined regions cyclin-N and cyclin-C are underlined.

Multiple sequence alignment of tomato cyclins revealed that most tomato cyclins contain a conserved cyclin core, which has a highly conserved domain cyclin N and/or a less conserved domain cyclin C (Figure 2). Pfam analysis indicated that almost all tomato cyclins have the cyclin N domain except U-type cyclins; C-, H- and J18-type only have cyclin C domain. Tomato U-type cyclins contain another potential cyclin domain, which is believed to play a role in phosphate signaling. In addition to the cyclin core, A- and B-type cyclins also contain a destruction box (D-box), which is involved in cyclin proteolysis by the ubiqutin-dependent proteasome pathway. D-type cyclins may have another motif called the PEST region, which is rich in Pro(P), Glu(E), Ser(S), and Thr(T) residues, and is a marker for unstable proteins.

**Figure 3.**
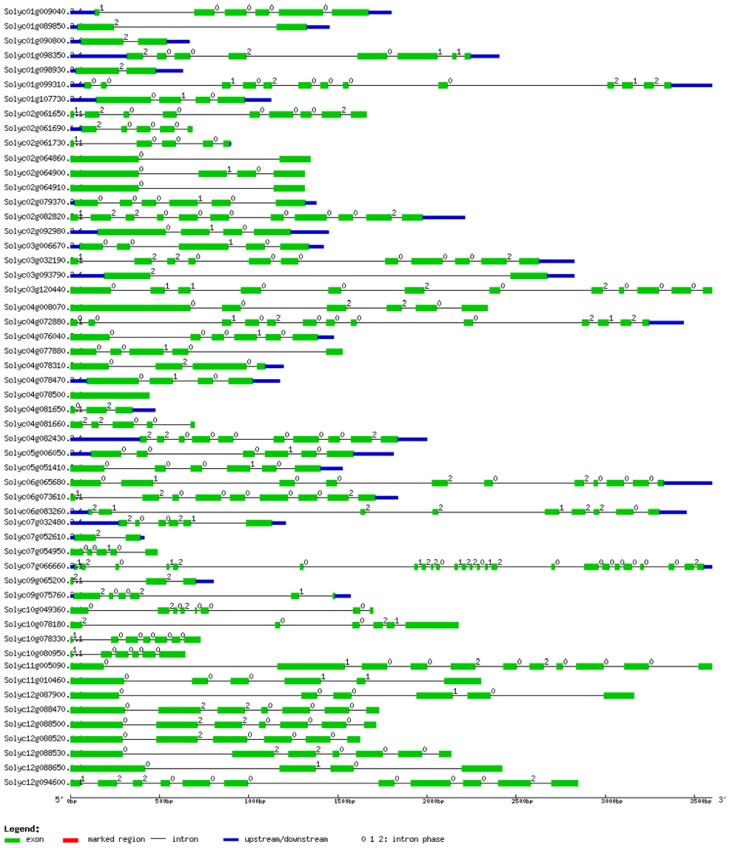
Gene structure of tomato cyclins generated from GSDS.

The presence of an existing cDNA with a known corresponding position on the genome was required for the determination of the number of exons and introns. Intron phase is preferentially associated with its own set of residues: phase 0 introns with lysine, glutamine, and glutamic acid before the intron, and valine after; phase 1 introns with glycine, alanine, valine, aspartic acid and glutamic acid; phase 2 introns with arginine, serine, lysine, and tryptophan. The intron positions are related to nucleotides, amino acid residues, and protein secondary structure. The intron numbers varied among different tomato cyclin members, and most of them ranged from 1 to 12. The data showed that one of tomato cyclins, *SlcyclinJ18*, has no intron. It is different from the *Arabidopsis cyclinJ18* gene, which contains 8 introns. Our data also shows that one tomato cyclin, *SlCycB3;1*, contain 24 introns, while its homolog *CycB3;1* from *Arabidopsis* only contains 22 introns (Figure 3); this means the tomato *SlCycB3;1* might possess more introns by DNA fragment insertion.

**Figure 4.**
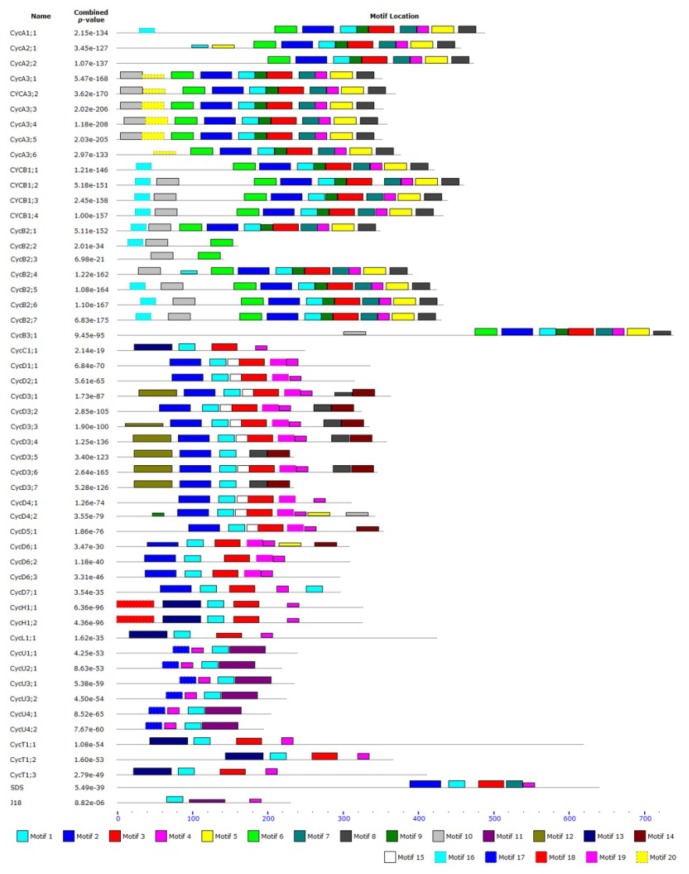
MEME software characterized conserved motifs of tomato cyclins. Different colors represent different motifs and each motif is represented by a box numbered at the bottom. The names of genes and combined P value are exhibited on the left side of the figure. Grey lines represent the non-conserved sequences; the length of protein can be estimated using the scale at the bottom. Motifs 2 and 6 are the cyclin-N domain; Motifs 1,3,7 and 9 are the cyclin-C domain; Motifs 11 and 17 are the U-type cyclin domain; Motifs 14 is the D-type PEST region.

Conserved motifs in cyclin genes were identified using the MEME motif search tool. Distinct motifs and their information were listed in Table 2. The locations of motifs matched well with the conserved regions revealed by multiple sequence alignment analysis. Twenty conserved motifs were identified in the tomato cyclin gene family (Figure 4). Motif 1, 2, 3 or 4 were found in most of the tomato cyclin family members, indicating that these conserved motifs may play critical roles in subfamily-specific functions. The same types of cyclins contain similar motifs. Other unknown motifs were also revealed by MEME motif search. Motifs 2 and 6 were mainly found in the *N*-terminal regions, while motifs 1, 3, 7 and 9 were found mainly in the *C*-terminal regions. Only U-type cyclin had motifs 11 and 17 cyclin domains and motif 14 may be the PEST region of d-type cyclin. Although the functions of some motifs are not yet clear, the presence of these conserved motifs certainly reflects functional similarities among tomato cyclins sharing these common motifs.

### 2.4. Chromosomal Localization and Gene Duplication

**Figure 5.**
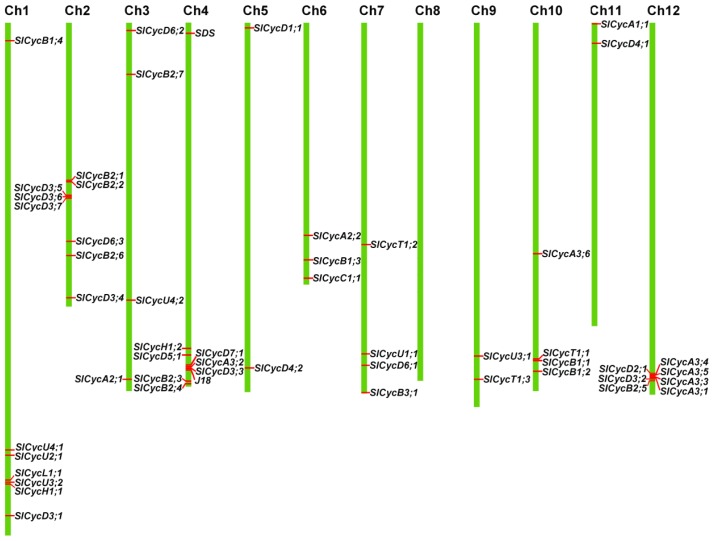
Genome-wide distribution of cyclins on tomato chromosomes. Gene identity numbers are provided. Chromosome numbers are indicated at the top of each bar.

To determine the genomic distribution of cyclin genes, their chromosomal positions were identified according to the SGN database. All of the cyclin genes are distributed on 11 chromosomes throughout the tomato genome. However, the number of cyclin genes on each chromosome varies widely. A maximum number of nine genes is present on chromosome 4, followed by eight genes on chromosome 2. On the other hand, no cyclin gene was present on chromosome 8. Two chromosomes, 4 and 12, have a group of cyclin genes in the vicinity of each other (Figure 5). Each type of cyclin gene except C-, L-, SDS and J18 was found distributed on at least two chromosomes. B-type cyclin genes are dispersed on up to eight chromosomes. Five of sixteen D-type cyclin genes are concentrated on chromosome 2, and four of nine A-type cyclin genes were distributed on chromosome 12. Chromosome 4 contained several types cyclin genes, such as A-, B-, D-, H-, SDS and J18 type, but chromosome 5 contained only one d-type cyclin.

**Figure 6.**
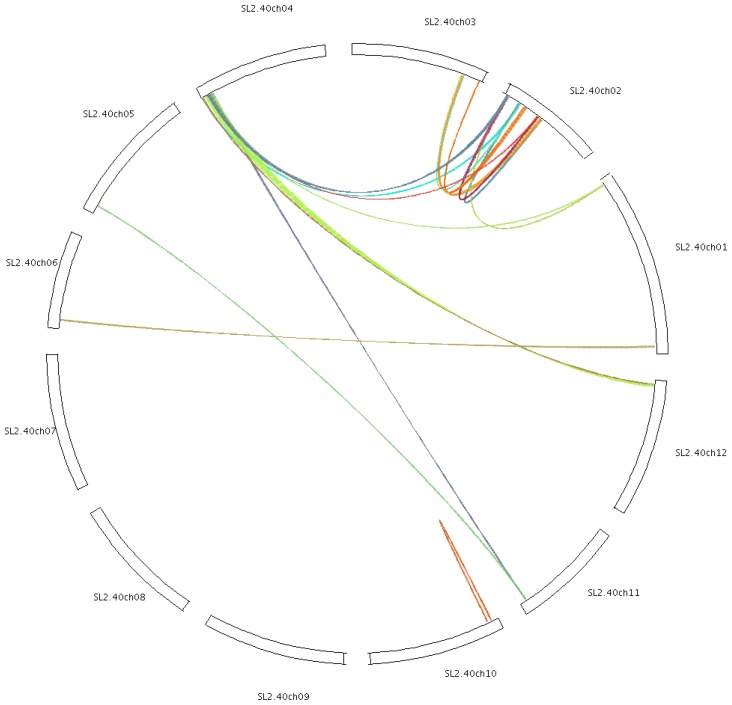
Depiction of segmentally duplicated cyclins on 12 tomato chromosomes.

Gene families are generated through either tandem duplication or large-scale segmental duplication during evolution [24]. Among tomato cyclin genes, 30 were found to be segmentally duplicated, which are located on duplicated segments on chromosomes 1, 2, 3, 4, 5, 6, 10, 11 and 12 (Figure 6). A maximum of nine cyclins are located in duplicated segments in chromosome 2, seven cyclins on chromosome 4, three cyclins on chromosomes 1 and 3, and two cyclins on chromosomes 10, 11 and 12. Duplicated segments on chromosomes 5 and 6 contain one cyclin. *SlcycB1;1* and *SlcycB1;2* were located on chromosome 10 and they were adjacent to each other. *SlcycA3;1* and *SlcycA3;2*, *SlcycB1;3* and *SlcycB1;4*, *SlcycB2;4* and *SlcycB2;5*, *SlcycB2;6* and *SlcycB2;7*, *SlcycD4;1* and *SlcycD4;2*, *SlcycD6;2* and *SlcycD6;3* were located on different chromosomes, respectively, and showed high sequence identities at protein level. We presumed that one of the genes derived from the other gene, and they underwent intra- or inter-chromosomal segmental duplication.

### 2.5. Organ-Specific Expressions of Tomato Cyclin Genes

Since gene expression patterns can provide important clues for gene function, we performed quantitative RT-PCR to characterize the gene transcription profiles of the tomato cyclin genes from different tissues including roots, stems, young leaves, flower, mature green fruit, breaker fruit and ripe fruit. Since it is hard to exhaustively describe the expression profiles of all tomato cyclin genes, only 14 members belonging to 10 types of tomato cyclins genes were selected to be checked.

**Figure 7.**
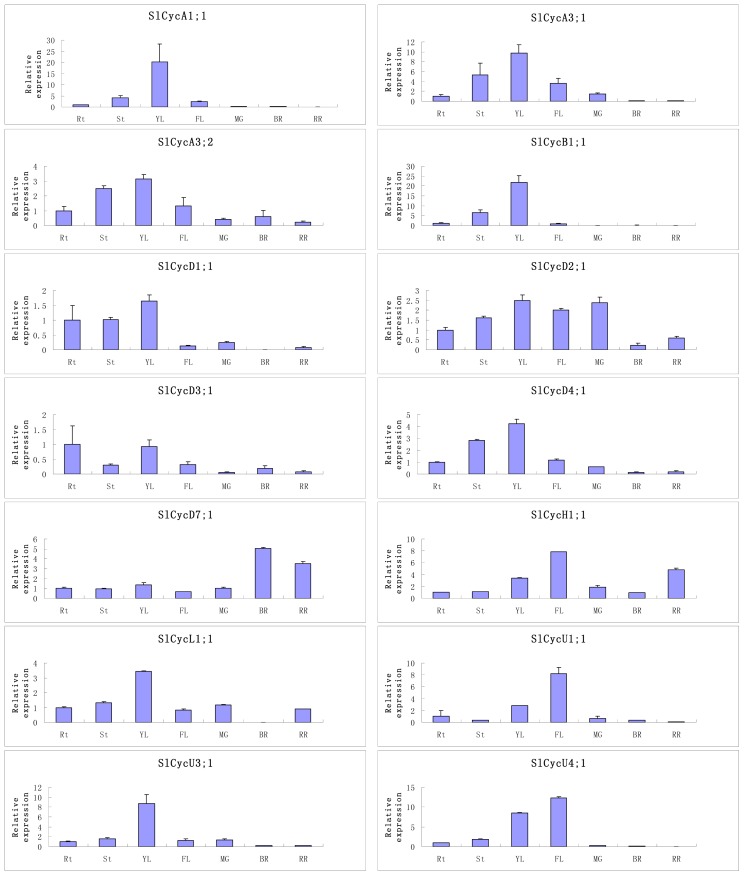
Expression patterns of tomato cyclin genes in different tissues or organs. Rt, root; St, stem; YL, young leaf; FL, flower; MG, mature green fruit; BR, breaker fruit; RR, ripe fruit. Error bars represent standard deviations for three replicates.

Previous studies have suggested that A-, B- and D-type cyclins were predominantly expressed in mitotically active organs: developing fruits, young leaves and roots [25]. As shown in Figure 7, the examined tomato A- and B-type cyclins genes were mainly expressed in young leaves and stems. Most examined D-type cyclins appeared to be constitutively expressed in all examined tissues. But during fruit development and ripening, most of the D-type cyclins show lower expression levels. *SlcycD1;1*, *SlcycD2;1*, *SlcycD3;1*, and *SlcycD4;1* showed the lowest transcriptional levels at breaker stage, and the transcripts of *SlcycD3;1* could not even be detected at this stage. Surprisingly, *SlCycD7;1* showed a higher expression pattern in fruit at breaker and red ripe stages, indicating that different tomato D-type cyclins might function in different ways to regulate tomato fruit growth and development, and the major function of *SlCycD7;1* might be related to tomato fruit ripening. Our results also showed that, the examined tomato H- and L-type cyclin genes are likely expressed in all checked tissues, with a relatively higher level in flowers and young leaves. *SlCycU1;1* and *SlCycU4;1* showed a higher expression level both in young leaves and flowers, whereas *SlCycU2;1* only showed higher expression level in young leaves. Like most of tested tomato A-, B-, and D-types cyclin genes, all examined tomato L- and U-type cyclin genes also showed lowest expression levels during fruit ripening. Our results also showed that a few tomato cyclin genes exhibit tissue-specific expression. For example, *SlCycB1;1* is expressed only in vegetative organs. The tissue-specific expression profiling of tomato cyclins might enable the combinatorial usage of the genes in transcriptional regulation of different tissues, whereas ubiquitously expressed tomato cyclins might regulate the transcription of a broad set of genes.

### 2.6. Expression of Tomato Cyclin Genes in Response to Exogenous GA

**Figure 8.**
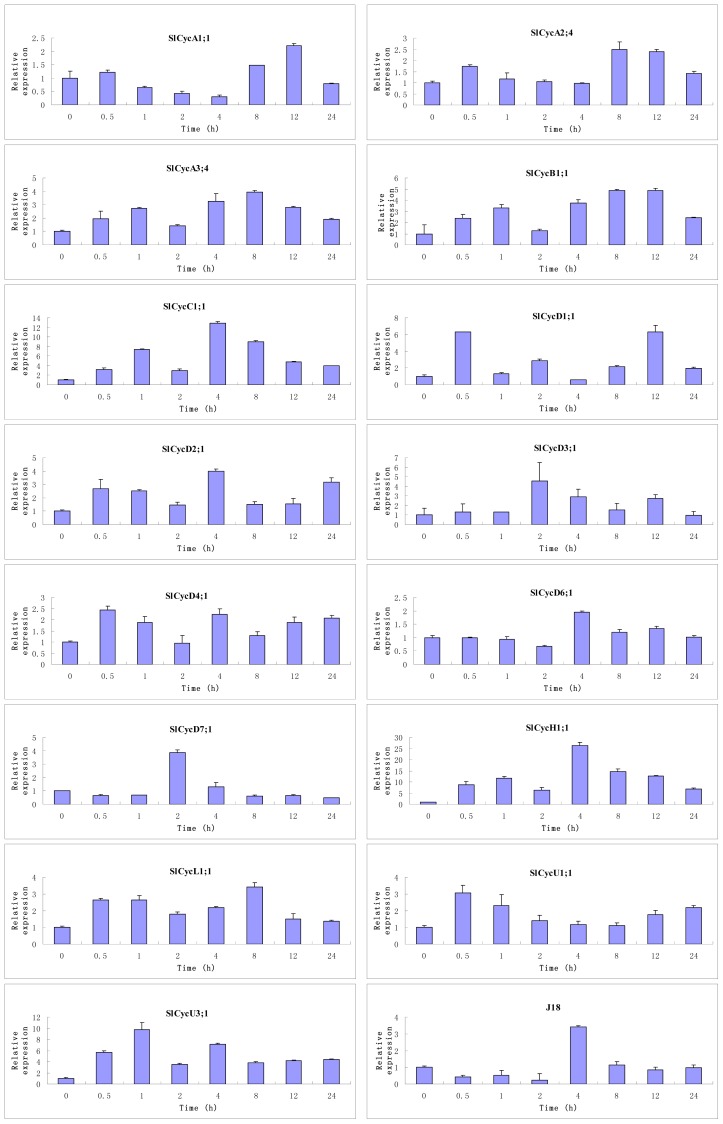
Expression profiles of the tomato cyclin genes after exogenous GA treatment. The relative transcript levels of these genes were analyzed over a 24 h period after 100 μM GA treatment.

Phytohormones are the major regulators of plant growth and development [26] and GAs are a class of phytohormones which can exert much influence on plant developmental processes, including stem elongation, leaf expansion, seed germination, flowering, sex expression, and leaf and fruit senescence [27]. GAs were sufficient to regulate the expression of cell cycle genes *cycA1;1* and *cdc2Os-3* in deepwater rice [22]. To determine which cyclin genes could respond to GA, we analyzed the expression patterns of a few cyclin genes followed with exogenous GA treatment. The results indicated that the expression of *SlCycA3;1*, *SlCycB1;1*, *SlCycC1;1*, *SlCycD2;1*, *SlCycD4;1*, *SlCycH1;1*, *SlCycL1;1*, *SlCycU1;1* and *SlCycU3;1* were GA induced, whereas the expression of *SlCycD7;1* and *J18* were GA suppressed. No obvious modification of the expression of *SlCycA1;1*, *SlCycA2;4*, *SlCycD3;1* and *SlCycD6;1* were observed after GA treatment, which indicated that these genes may not be directly regulated by GA (Figure 8).

## 3. Discussion

### 3.1. Cyclin Gene Family and Their Structures

Previous research has indicated that each cyclin plays a distinct role in cell cycle progression and cell division [11,13,14,19]. Cyclins can be divided into several classes according to their sequence similarity, expression patterns and protein activity during the cell cycle [28–32]. The numbers of cyclins in plant genomes are variable; it has been reported that the rice genome contains 49 cyclins and they can be classified into 9 types, including A-D-, F-, H-, L-, P-, and T-type [4]. Maize genome contains 59 cyclins, which can only be classified into 6 types, including A-D-, F-, T-, and SDS-type [33]. 45 cyclins in the poplar genome were identified and can be classified into 7 types, including A-D-, Q-, T-, and Z-types [7]. The *Arabidopsis* genome contains 50 cyclins, which can be classified into 10 types, including A-D-, H-, L-, P-, T-, J18-, and SDS-type [3]. In our research, 52 cyclin genes in tomato genome were identified; as in *Arbidopsis*, these cyclins can be classified into 10 types.

Since all of these reported plant genomes contain A-, D- and T-type cyclin genes, it may implied that these three types of cyclins are more conserved throughout the plant species. *Arabidopsis* and tomato shared the same 10 types of cyclins, and the number of cyclins in these two genomes is similar. This illustrates that the cyclin gene family in these two genomes is relatively conserved. Furthermore, a closer genetic relationship between *Arabidopsis* and tomato than any other reported plants was also confirmed.

Protein sequence alignment and structure analysis shows considerable conservation and specific motifs in tomato cyclins (Figures 2 and 4). Similar to other cyclin families in plant species, almost all tomato cyclins contain a cyclin N domain except U-type cyclins, and C-, H- and J18-type cyclins only contain a cyclin N domain. This means that the N domain is more conserved than the C domain, and this was further confirmed through chromosome distribution analysis and cyclin motifs character analysis.

### 3.2. Duplication of Tomato Cyclin Genes

Gene duplications are one of the primary driving forces in the evolution of genomes and genetic systems [34]. The dramatic variations of most gene families in family size and distribution are affected by tandem duplications and segmental duplications [24]. Studies of *Arabidopsis* reveals that its genome contains a lot of large segmental duplications that originated from continuous polyploidy events and has been subjected to scrambling by chromosomal rearrangements [35–38]. In tomato, most of the duplications were concentrated on chromosome 2 and 4. It seems that duplication and subsequent expansion of cyclin genes occur frequently throughout evolution. These results shed light on the evolution process of the tomato genome. These results also indicate the presence of similar or overlapping functions among all segmentally duplicated cyclin genes, although their remarkable differences in amino acid sequences were observed. In this research, we also find that the expression pattern of tandem duplicated genes is highly similar.

### 3.3. Organ-Preferential and GA Responsive Expression Profiles of Tomato Cyclin Genes

Cyclin genes that show similar expression profiles during various developmental stages may have similar functions. According to the expression profile of tomato cyclins examined in this study, we can divide them into 3 different classes. The first class includes most of tomato cyclins, which are highly expressed in young leaves. The second class cyclin genes are mainly expressed in flowers. The third class cyclin genes transcripts can be detected in fruit at the breaker stage. It is interesting that most closely related cyclin genes show similar patterns of expression, such as *SlCycA1;1*, *SlCycA3;1* and *SlCycA3;2; SlCycU1;1* and *SlCycU4;1*, suggesting possible functional redundancy and conservation among these similar members.

Surprisingly, some cyclin genes of tomato and *Arabidopsis* within the same clade in phylogenic analysis showed different expression patterns. For example, *AtCycD7;1* was not detected in any of the *Arabidopsis* tissues or organs, whereas *SlCycD7;1* was detected in all of the tomato tissues examined, and showed a relative higher expression level in breaker fruit. *AtCycU4* was especially expressed in root, while *SlCycU4* showed a higher expression level in flower. These results suggest that homologous cyclin genes of different plants may have same function in the cell cycle, whereas the roles they played in different plants might be different. Understanding expression patterns in different tissues is the first step to clarify the function of cyclin genes. Therefore, cyclin genes with specific expression patterns could be the focus of functional studies in the future.

Cell expansion and cell division were also regulated by several plant hormones. It has been reported that GAs play important roles in regulating the transcription of several cell cycle genes [21,39,40]. In this research, we investigated the expression profiles of several cyclin genes following the treatment of exogenous GA. The transcripts of most tested tomato cyclins genes were elevated after GA treatment, differently, the transcription of *SlCycD7;1* and *J18* were obviously reduced, and only a few cyclin genes were not obviously affected (Figure 8). Previous studies have shown that GA can promote plant growth through cell expansion by stimulating the destruction of growth-repressing DELLA proteins, which can restrain cell cycle activity through enhancing the accumulation of cell cycle inhibitors. In this paper, we showed that GA can regulate the cell cycle through its influence on some cyclin genes’ transcription. It might be very interesting to uncover the regulatory mechanism underlying these processes.

## 4. Materials and Methods

### 4.1. Plant Materials

Tomato (*Solanum lycopersicum* L. cv Ailsa Craig) plants were grown in a glasshouse with the a 16 h light/8 h dark cycle. Samples of roots, stems, leaves, flowers and fruits were collected in adult plants, immediately frozen in liquid nitrogen and stored at −80 °C until use. In order to investigate the responses of the tomato cyclin genes to GA, six-week-old seedlings were sprayed with 100 uM GA, and the control plants were sprayed with water. Then the shoot samples (including leaves and stem) were harvested at 0, 0.5, 1, 2, 4, 8, 12 and 24 h later. Samples of control plants, which were sprayed with water, were also collected as the GA treatment plants. These samples were also immediately frozen in liquid nitrogen and stored at −80 °C. For all experiments, three biological samples were collected for further analysis.

### 4.2. Identification of Putative Tomato Cyclin Genes

The full length cDNA sequences of *SlCycA1;1*, *SlCycA2;1*, *SlCycA3;1*, *SlCycB1;1*, *SlCycB2;1*, *SlCycD3;1*, *SlCycD3;2* and *SlCycD3;3* were obtained from NCBI as reported previously [18,29]. To obtain more cyclin gene sequences in tomato, the cDNA sequences of the above genes were subjected to BLASTN searches in the SOL Genomics Network (SGN) [41], NCBI [42] and DFCI [43] databases. Almost all of the Arabidopsis cyclins genes were reported previously, the sequences of these genes were extracted from NCBI database, and then they were used to do TBLASTN searches in the SGN database with the E-value cutoff set as le-10. To get more information on tomato cyclin genes, name search, using cyclin as a keyword in these databases was also conducted. All the protein sequences were analyzed in the Pfam HMM database [44] to find cyclin-N and cyclin-C domains. Overlapping genes were removed using a complete sequence alignment method in MEGA 5.0 [45] to obtain the final set of non-overlapping cyclin genes in tomato.

### 4.3. Phylogenetic Analysis of Tomato Cyclins

Sequence alignment of multiple cyclins was performed using MEGA 5.0 [45], and an unrooted phylogenetic tree was constructed using the neighbor-joining (N-J) method with the pairwise deletion option selected and the passion correction set for the distance model. Phylogenetic relationships were analyzed by conserved structural alignments. Bootstrap analysis was performed using 1000 replicates.

### 4.4. Structure Analysis of Cyclins in Tomato

The cDNA sequences and their corresponding genomic DNA sequences of tomato cyclins were obtained from SGN database, then they were analyzed using the GSDS (Gene Structure Display Server) software [46]. To identify conserved cyclin motifs, the protein sequences of tomato cyclins were merged using the GENESTUDIO software that we download from the website (http://www.genestudio.com/), and then the protein sequences were analyzed using the MEME program [47]. The MEME program was employed using the following parameters: number of repetitions-zero or one, maximum number of motifs-20, optimum motif width set to >6 and <50.

### 4.5. Chromosomal Localization and Gene Duplication Analysis of Tomato Cyclins

The chromosomal distribution of putative tomato cyclins were identified by identifying their chromosomal position given in the SGN database.

To find potential duplicated tomato cyclin genes, MCScanX software (http://chibba.pgml.uga.edu/mcscan2/) [48] was used. All tomato cyclin genes were compared against themselves and others using BLASTp program, with criterion of tabular format (-m 8) –b 5 –v 5 and e-value of 1e-5. The resulting blast hits were incorporated along with chromosome coordinates of all protein-coding genes as an input for MCScanX under default criterion. The result was analyzed using Perl script.

### 4.6. RT-PCR and Real-Time qRT-PCR

In order to study the expression patterns of the cyclins in various tissues, total RNAs were isolated from roots, stems, leaves and fruits at the stage of mature green (MG), breaker (BR) and red ripe (RR) using TriZol reagent (Invitrogen, Gaithersburg, MD, USA), according to the manufacturer’s instruction. The total RNA samples were treated with RNase-free DNase I to remove residual genomic DNA. About 3 μg of the DNase I treated total RNA was used for first-strand cDNA synthesis with M-MLV reverse transcriptase and Oligo (dT)_25_; the product was diluted to a final volume of 200 μL. The primers used for the real-time PCR were designed by primer premier 5.0. Real-time PCR was performed with LightCycler 480 instrument. Each reaction contained 5 μL SYBR Premix, 3 μL cDNA samples, and 0.5 μL of 10 μM gene specific primers in a final volume of 10 μL. The thermal cycle was as follows: 95 °C for 5 min, 40 cycles of 95 °C for 10 s, 58 °C for 15 s and 72 °C for 20 s. Three technical replicates were performed for each sample. Tomato actin gene (GenBank ID: BT012695) was used as an internal control. All the primers used were listed in Table 3.

## 5. Conclusions

In conclusion, we have presented an expression profile of 52 tomato cyclin genes along with an account of their phylogenetic relationships with *Arabidopsis* cyclin genes. The result indicates that groups of genes that show similar expression profiles during various developmental stages may or may not have similar functions. Protein sequence alignment and structure analysis show considerable conservation and specific motifs in tomato cyclins. Duplication and subsequent expansion have occurred frequently through the evolution of the tomato genome. The transcription of some cyclins can be induced by GA, suggesting that they may play a potential role in GA response. Therefore, these data would be useful in selecting candidate genes for functional validation in relation to various aspects of tomato vegetative growth and reproductive development.

## Figures and Tables

**Table 1.** List of cyclin genes identified in tomato. Predicted genes and related information include sequenced ID, chromosome locations and protein length.

Gene name	Annotated CDS	Chr. No.	Protein length (aa)
*SlCycA1;1*	Solyc11g005090.1.1	11	490
*SlCycA2;1*	Solyc03g120440.1.1	3	458
*SlCycA2;2*	Solyc06g065680.2.1	6	475
*SlCycA3;1*	Solyc12g088530.1.1	12	354
*SlCycA3;2*	Solyc04g078310.2.1	4	371
*SlCycA3;3*	Solyc12g088520.1.1	12	355
*SlCycA3;4*	Solyc12g088470.1.1	12	360
*SlCycA3;5*	Solyc12g088500.1.1	12	354
*SlCycA3;6*	Solyc10g049360.1.1	10	378
*SlCycB1;1*	Solyc10g078330.1.1	10	423
*SlCycB1;2*	Solyc10g080950.1.1	10	462
*SlCycB1;3*	Solyc06g073610.2.1	6	440
*SlCycB1;4*	Solyc01g009040.2.1	1	435
*SlCycB2;1*	Solyc02g061650.1.1	2	351
*SlCycB2;2*	Solyc02g061690.1.1	2	162
*SlCycB2;3*	Solyc04g081660.1.1	4	142
*SlCycB2;4*	Solyc04g082430.2.1	4	393
*SlCycB2;5*	Solyc12g094600.1.1	12	425
*SlCycB2;6*	Solyc02g082820.2.1	2	434
*SlCycB2;7*	Solyc03g032190.2.1	3	431
*SlCycB3;1*	Solyc07g066660.2.1	7	739
*SlCycC1;1*	Solyc06g083260.2.1	6	250
*SlCycD1;1*	Solyc05g006050.2.1	5	337
*SlCycD2;1*	Solyc12g087900.1.1	12	316
*SlCycD3;1*	Solyc01g107730.2.1	1	364
*SlCycD3;2*	Solyc12g088650.1.1	12	325
*SlCycD3;3*	Solyc04g078470.2.1	4	336
*SlCycD3;4*	Solyc02g092980.2.1	2	359
*SlCycD3;5*	Solyc02g064860.1.1	2	235
*SlCycD3;6*	Solyc02g064900.1.1	2	346
*SlCycD3;7*	Solyc02g064910.1.1	2	235
*SlCycD4;1*	Solyc11g010460.1.1	11	312
*SlCycD4;2*	Solyc05g051410.2.1	5	344
*SlCycD5;1*	Solyc04g076040.2.1	4	355
*SlCycD6;1*	Solyc07g054950.1.1	7	310
*SlCycD6;2*	Solyc03g006670.2.1	3	311
*SlCycD6;3*	Solyc02g079370.2.1	2	297
*SlCycD7;1*	Solyc04g077880.1.1	4	298
*SlCycH1;1*	Solyc01g099310.2.1	1	328
*SlCycH1;2*	Solyc04g072880.2.1	4	327
*SlCycL1;1*	Solyc01g098350.2.1	1	426
*SlCycU1;1*	Solyc07g052610.2.1	7	241
*SlCycU2;1*	Solyc01g090800.2.1	1	220
*SlCycU3;1*	Solyc09g065200.2.1	9	237
*SlCycU3;2*	Solyc01g098930.2.1	1	226
*SlCycU4;1*	Solyc01g089850.2.1	1	206
*SlCycU4;2*	Solyc03g093790.2.1	3	196
*SlCycT1;1*	Solyc10g078180.1.1	10	621
*SlCycT1;2*	Solyc07g032480.2.1	7	368
*SlCycT1;3*	Solyc09g075760.2.1	9	413
*SDS*	Solyc04g008070.1.1	4	641
*J18*	Solyc04g078500.1.1	4	231

**Table 2.** Details of the putative motifs.

Motif	Motif length (AA)	Best possible match
1	21	LQLLGVTCLLLAAKYEEIxVP
2	41	QKDVNESMRGILVDWLVEVHDKYKLxPETLYLAVNYIDRFL
3	33	VLRMEKLVLNTLKWRMTVPTPYTFLRRFLKAAQ
4	15	PSxIAAAAIYLARFT
5	29	HPWSLTLEHHTGYSESQLKECVLLIVDLH
6	29	DDAKNPLACVEYVEDIYAYYKKMEIEKRR
7	21	LEFLSFYLAELCLLEYECLKF
8	23	KLTAVRRKYSSHKFKCVALLGPP
9	15	VEDFCYITDNAYTKK
10	29	PPVRVTRPATRKFAAQMASQLQQPNKKRV
11	47	AYYAKVGGITTREMNKLEVDFLFGLGFQLHVNVTTFESYCSYLEKEM
12	50	CKEDPLDEGDLGGGYHSDERNWNVKKISPLLECDMFWEDGEVETLLSKEK
13	50	NSPSRKDGIDVEEEQHLRKFYCFFLQDLGIRLKFPQKTIATALILCHRFY
14	29	GVIDAYFSSESSNDSWVVASSVSSLPEPQ
15	15	DLQVEDAKFVFEAKT
16	20	GGERGRNRRALGDINQNLVG
17	21	FHGLRAPNISIQSYLERIFKY
18	49	MADFVTSTHRTKWIFTPQDIKHKYKVANHRAKQALEKYGTTRMEVDIDG
19	21	LLRRAEQLILSTITDIRFLEY
20	29	VLSEIQDLCNVGINQIEDKVFVSEPLRPK

**Table 3.** List of primers used for Real-time qRT-PCR.

Gene	Forward primer sequence	Reverse primer sequence
*SlCycA1;1*	ATGACAACCCAGAATAAGCG	ACCGAGTTCCGAGCAGAG
*SlCycA2;4*	TTGGGTTCATCAGGAGGACT	CTTCGCTTAGGCTGTTGAGA
*SlCycA3;1*	CTAAGAAAAGAGCAGCAGAAGCA	GATTCCTTATCTTTTTCAGCAACAG
*SlCycA3;2*	GCTCCACAGGAACAACGAA	CCACAACCCTTTTCACCTTC
*SlCycA3;4*	AACCCACGAATCCAAACG	CCAAGGAGTGAAGATGCTGA
*SlCycB1;1*	GTATCTCGCCCCGTAACAAG	TCTCCTCAGGTTTTGGCTTT
*SlCycC1;1*	AAGTTCTGGAAGCCCTAAACTATTA	GAATTAGGTCCATCTTATAGGTGTCA
*SlCycD1;1*	CTGCTACTTCTTCTTCAAATCCA	CGGAGAAACAGTCGGAGTAA
*SlCycD2;1*	GTGATGGCTCAGGGGTTG	GTTATGTGTTTGTGGGGTGC
*SlCycD3;1*	CTGCCAAAGCCTCAAGCG	CAGTGGAGCTAGTGTCATTCGC
*SlCycD4;1*	GCAGTGGCAATGTCTGTTTC	CCATTGGGAGTTTGAGGC
*SlCycD6;1*	GATGGAGACGACGATGACGA	TGATTGCGAATGGAAGAGTGA
*SlCycD7;1*	AGATGGAGAGTTTGCTTTGTGAT	AAAATCTTAAAAGCCTCTTCACAAT
*SlCycH1;1*	ACCTGGGCAGTTGGCATT	CGTCTTTGGAAGTCGGAGTC
*SlCycL1;1*	CGTAGATTTCAAGTGCCCCTC	TATTTTTGCTTTTGGAAGACTGTAA
*SlCycU1;1*	CGGACGATGTAGCCACCC	CCTGCCCCGTTGCTCAT
*SlCycU3;1*	ATTGTCTTCTTGCCCCTTTT	TCAGGTTCAGGTGCCAAAG
*SlCycU4;1*	TCAAAATCTGATGCCGAAAC	CGAAACAACAAGGGCTACAA
*J18*	GATCGAGAATGAAGAAGGAGGT	GATAGAATCGGTTGGCAAAAGT
*Actin*	GTCCTCTTCCAGCCATCCAT	ACCACTGAGCACAATGTTACCG

## Abbreviations

RT-PCR: reverse transcription polymerase chain reaction
GA: gibberellic acid
